# Stress induced delamination of suspended MoS_2_ in aqueous environments†

**DOI:** 10.1039/d2cp02094g

**Published:** 2022-07-29

**Authors:** Michal Macha, Mukeshchand Thakur, Aleksandra Radenovic, Sanjin Marion

**Affiliations:** Ecole Polytechnique Federale de Lausanne (EPFL) Lausanne Switzerland michal.macha@epfl.ch aleksandra.radenovic@epfl.ch sanjin.marion@imec.be

## Abstract

Applying hydrostatic pressure with suspended 2D material thin membranes allows probing the effects of lateral strain on the ion and fluid transport through nanopores. We demonstrate how both permanent and temporary delamination of 2D materials can be induced by pressure and potential differences between the membrane, causing a strong mechanosensitive modulation of ion transport. Our methodology is based on *in situ* measurements of ionic current and streaming modulation as the supporting membrane is indented or bulged with pressure. We demonstrate how indirect measurements of fluid transport through delaminated MoS_2_ membranes is achieved through monitoring streaming current and potential. This is combined with TEM images of 2D material membranes before and after aqueous measurements, showing temporary delamination during mechanical or electrical stress. The obtained results allow a better understanding of measurements with supported 2D materials, *i.e.* avoiding misinterpreting measured data, and could be used to probe how the electrical field and fluid flow at the nanoscale influence the adhesion of supported 2D materials.

## Abstract 2

Mechanical and electrical stress can lead to the temporary and permanent 2D membrane delamination and consequent strong mechanosensitive modulation of ion transport measured *in situ*.

## Graphical abstract

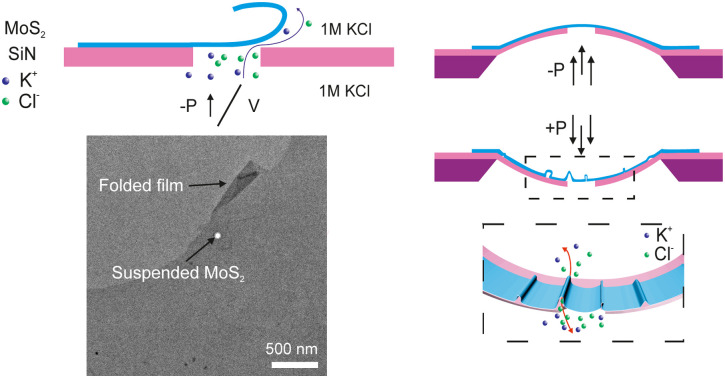

## Introduction

The unique properties of atomic thickness, mechanical and electric properties^1–6^ as well as rapid advancements in 2D materials synthesis methods^7,8^ have made 2D-material-based devices a platform of choice to study nanoscale physics in aqueous environments.^9^ Suspended, nanoporous 2D-membranes are used in applications such as biosensing,^10^ DNA translocation,^11^ osmotic energy harvesting,^12–14^ water desalination^15^ and gas filtration.^16^ In most nanofluidic experiments, the application of electrical fields is used as a basic tool to probe the ion transport properties of the system.^17,18^ Lately however, the use of hydrostatic pressure as an additional probe has been found to be critically important to study the nonlinear coupling of ion transport with fluid flow^19,20^ as well as a tool to probe proper wetting behaviour.^21^

It has been established, that applying high voltage can be potentially damaging to the 2D material as it can cause membrane breakdown^11,22^ and delamination through electrolyte intercalation.^23,24^ Even though the strain-induced wrinkling and delamination was thoroughly studied,^25–27^ it is not fully explored how applying hydrostatic pressure can influence the membrane performance and 2D film adhesion to the substrate in an aqueous environment. It was shown, that surface-related phenomena such as material damage, delamination or nanobubbles^28^ can exhibit nonlinear current signals,^20,21,23^ analogous to those reported as ionic coulomb blockade.^29^ Thus, even though MoS_2_ was proven stable at working pressure of up to 3.5 bar,^20,30^ further investigation of the application of both electrical fields and pressure is crucial to uncover adhesion-related artifacts, help understand the 2D-nanofluidic system and bring insights into designing an artifact-free 2D-material platforms.

In this work we are using a symmetrical hydraulic pressure based setup (*i.e.* with the pressure applied to the backside, frontside or both sides of the membrane simultaneously) to investigate the MoS_2_/membrane adhesion behaviour during nanofluidic experiments.^20,21^ We investigate the influence of applying high voltages (including voltage-mediated pore-drilling protocols) on the reversible 2D film delamination. We demonstrate how the membrane deformation through applied hydrostatic pressure can lead to adhesion defects such as MoS_2_ wrinkling and flapping manifested through extremely non-linear current signals and unstable streaming currents. With transmission electron microscopy (TEM) images taken before and after the measurements we showcase the reversibility of the delamination processes. Finally, by analyzing the asymmetric and unstable system behaviour under pressure we show how to properly identify and study membrane properties. The experimental methodology presented here enables to uncover the adhesion issues and brings a deeper understanding of nanoscale physics of suspended atomically thin films in aqueous solutions and their ion transport behavior under experimental stimuli.

## Results and discussion

### Voltage-driven delamination of MoS_2_

**Fig. 1:**
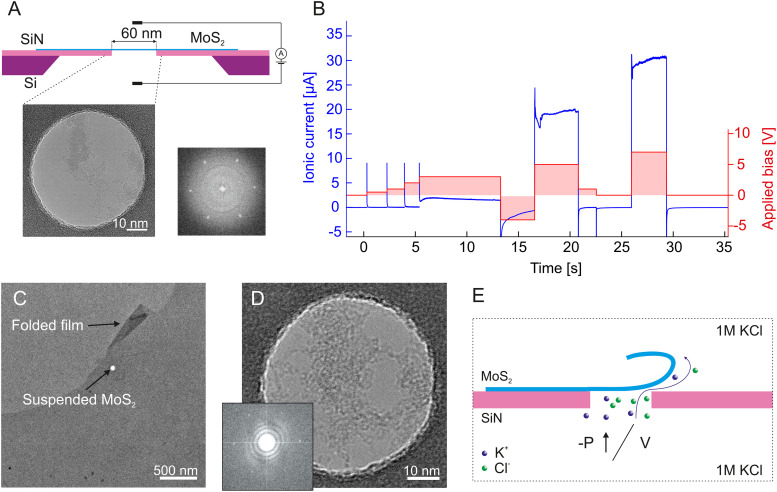
Temporary delamination of MoS_2_ during nanofluidic measurements. (A) Schematic description of the measurement geometry with a TEM image of suspended MoS_2_ and the corresponding FFT. The experimental setup and details match those described in ref. 20 and 21, including the transfer method for MoS_2_ and pressure setup schematics. (B) Measured values of the ionic current (blue, left axis) and applied potential difference (red, right axis) on a MoS_2_ sample *versus* time. Panels C and D show the same substrate as in panel A imaged after the ionic measurement in liquid (panel B), indicating that the MoS_2_ has survived the liquid immersion and measurement, and the presence of a “flap” near the aperture (panel C). In the proposed model, applied high voltage (or pressure) can cause intermittent delamination of MoS_2_ leading to ionic conduction through a created fluidic pathway (E).

We have used an atomically thin MoS_2_ membrane, irradiated with Xe ions and suspended over the Si/SiN aperture (see Materials and methods section) to probe the adhesion properties in an electrolyte solution. The MoS_2_ membrane was placed in the flowcell adapted to apply pressure gradients and test wetting of nanopores^20,21^ (Fig. 1A). Degassed 1 M potassium chloride (KCl) solution was used as an electrolyte on both *cis*- and *trans*-sides of the MoS_2_. A typical experiment with freestanding MoS_2_ membranes starts with probing the membrane conductance with voltage sweeps and ensuring proper wetting of the system through the application of hydrostatic pressure (*i.e.* achieving stable values of membrane resistance and capacitance expected of monolayer MoS_2_ suspended over the Si/SiN aperture^21^). Our wetting protocol involves flushing with a degassed electrolyte solution and applying hydrostatic pressure to both sides of the membrane (*i.e.* compression pressure) to reabsorb any potential, obstinate vapor bubbles back into the solution as reported previously.^21^

*In situ* nanopore creation is the crucial step of substrate preparation which can compromise the sample membrane. We have observed that this step, often regarded as robust and facile, can introduce crucial MoS_2_ delamination issues. The most commonly used method is the electrochemical reaction (ECR) drilling protocol.^11,17,31^ A methodology widely used in the nanopore research field to create pores *in situ* by applying gradually increasing high electric fields.^22^ A constant or periodic application of high potential differences between the two sides of the membrane is expected to cause defect expansion through an ECR and eventually form a nanopore of controlled dimensions.^11,22^ In an attempt to use high voltages to produce nanopores from pristine monolayer MoS_2_ membranes, we have applied voltages up to several volts. In some cases, we noted that after such applications, the resistance of the sample would afterward return to its original value. An example of such a measurement with a pristine MoS_2_ membrane is shown in Fig. 1B. Typically, the ECR and subsequent MoS_2_ pore formation is expected to start occurring at applied voltages as low as 0.75 V in MoS_2_,^11^ with drilling protocols reported up to 7 V for graphene.^31^ Higher voltages are in general expected to increase the probability of drilling a pore in ultrathin membranes.^22^ In our case, we applied potential differences as high as 7 V and after comparing the substrate before and after experiments in TEM (Fig. 1C and D) we found not only that the intact MoS_2_ surface has survived the ECR drilling protocol, but also a presence of a folded layer in close vicinity to the suspended area (Fig. 1C) indicating that the MoS_2_ has shifted in its placement on the substrate.

As observed previously in graphene, applying a high electric field can cause direct membrane delamination from the substrate^23^ which can impact membrane conductance (Fig. 1E). Such a loss in adhesion was found to occur after exceeding a threshold transmembrane voltage of 0.25–0.5 V depending on factors such as 2D film surface roughness, existing defects or surface folds.^23^ The process was found to be reversible *i.e.* delaminated 2D material relaminates after the transmembrane potential difference is removed and the electrolyte intercalation between 2D film and substrate is no longer energetically favorable.^23^ A possible mechanism used in the literature for similar effects would involve local Joule heating at the pore causing liquid superheating producing explosive nucleation of a vapor bubble.^32,33^ In our case MoS_2_ delamination *via* local bubbling on the MoS_2_/SiN interface is unlikely as we use an electrolyte solution that is undersaturated with gas and the starting ionic current through the pore is too small to induce significant Joule heating. On the other side, ionic-current induced pore enlargement is also not anticipated as it is reported to occur at low- and moderate applied electric fields and would cause permanent pore etching.^34^ We do confirm the delamination hypothesis by observing the reversible increase in ionic conductance while applying high voltage and measuring a nonlinear IV curve shape (Fig. S1, ESI†). This is in accordance to reported studies of voltage-driven graphene delamination^23^ triggered by an electric force. We provide additional proof with TEM images showcasing the survival of intact suspended MoS_2_ after the experiment (Fig. 1E). We hypothesize a forming of a reversible conductive pathway through locally delaminated MoS_2_ (Fig. 1E) which then collapses back when there is no external stimuli.

### Pressure driven, irreversible delamination of MoS_2_

We have observed that delamination may in some cases occur without high voltages but with applying pressure from the backside of the membrane (causing membrane *bulging*). We see the opening of a conductive channel in the membrane manifested through rapid increase in conductance as the pressure crosses a critical value due to the membrane being bulged (see Fig. S2, ESI†). The intact character of suspended MoS_2_ area is seen with multiple samples imaged post-experiment (see Fig. S3, ESI†), indicating liftoff of the MoS_2_ monolayer from the substrate and subsequent reattachment after removing from the flowcell environment (see Materials and methods section). The exact pressure value at which delamination happens varying from sample to sample (ranging from approx. 2–3 bar for 20 nm thick SiN membranes with square window sizes of 20 μm by 20 μm). The possible cause may be the difference between the MoS_2_ monolayer film area (*i.e.* the size of a MoS_2_ single crystal) transferred over the SiN aperture, varying between substrates, and batch-to-batch differences in the roughness of SiN surface.^35^ This is an inherent issue with currently used state-of-art thin film transfer techniques. The normal force on the suspended MoS_2_ can be estimated from the diameter of the pore of *d* = 60 nm to be about 1.5 nN at 3 bar of pressure. This pressure causes a force that is an order of magnitude smaller than typical forces applied in mechanical indentation experiments with atomic force microscopy^30,36^ and is not expected to compromise the mechanical stability of the suspended film. We conclude that hydrostatic pressure applied from the backside can therefore lift-off the MoS_2_ – causing *in situ* delamination with possible subsequent reattachment of the 2D material to the surface in a different condition (*i.e.* after removing the substrate from aqueous solution).

### Pressure driven, reversible delamination of MoS_2_

**Fig. 2:**
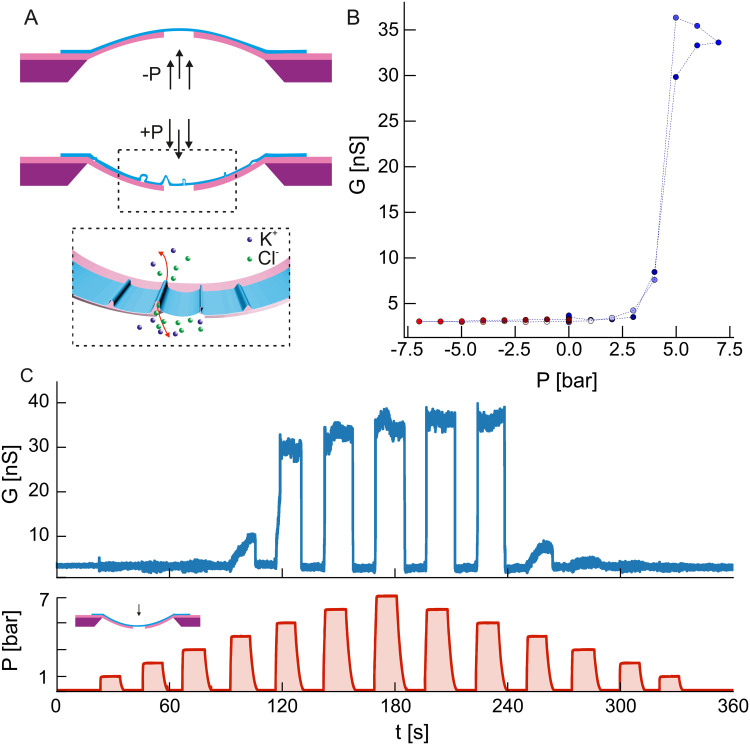
Delamination of suspended MoS_2_ due to pressure induced surface indentation. (A) Toy model demonstrating bulging (top) and indentation (bottom) of the membrane under positive and negative hydraulic pressure gradients. The bottom inset shows the proposed mechanism for the formation of conductive channels between two sides of the membrane due to local wrinkling and detachment of the MoS_2_. (B) Conductance *G* of the sample was obtained through ionic current measurements at 100 mV at different values of the pressure gradient *P* applied to the membrane. The measurement protocol involved subsequent measurements at different pressure values followed by a measurement at *P* = 0 bar to confirm that the baseline value has not changed. Panel (C) shows the time traces of the pressure *P* and conductance *G* as shown in panel b. Note that only positive pressure gradient values are shown to emphasize the abrupt increase in conductance seen in panel B.

**Fig. 3:**
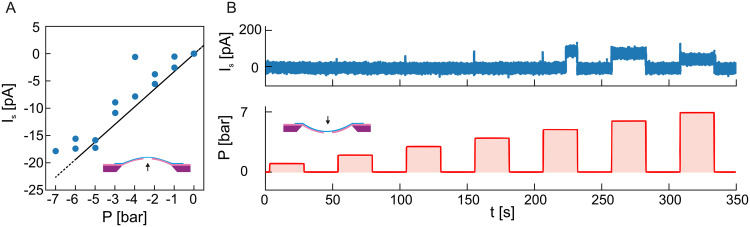
Streaming measurements on suspended MoS_2_ during delamination. (A) Streaming current *I*_s_ as measured on the sample shown in Fig. 2b and c during the membrane bulging state. No external potential was applied to the sample (closed circuit measurement). (B) Anomalous increase of streaming current *I*_s_ for positive pressures matching the delaminating state in Fig. 2b and c. The top panel show the time trace of the measured streaming current *I*_s_, while the bottom panel shows the applied pressure gradient *P*.

**Fig. 4:**
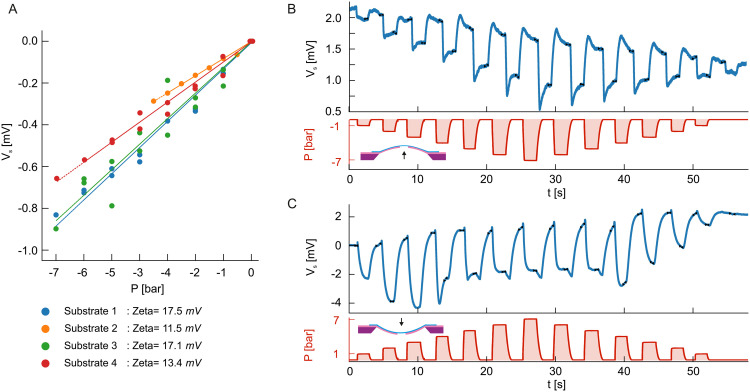
Streaming measurements on suspended MoS_2_ during delamination. (A) Streaming potential *V*_S_ measurements obtained for four different samples showing analogous behaviour as in Fig. 2b and c. Measurements were done in the open circuit configuration. A linear fit gives the values of the apparent *zeta* potential for the membrane. (Note that the measurement protocol follows the same approach as before with intermediary measurements at *P* = 0 to check for baseline drift, and two measurements at each intermediary pressure value to check for hysteresis after the maximal pressure value was applied) Time traces of the streaming potential *V*_S_ and applied pressure gradient for Sample 1 are shown in panels B and C. Panel (B) shows measurements of the streaming potential during the bulging case, while panel C during the delaminating case.

Interestingly, the same pressure applied from the membrane's backside does not yield symmetrical results when we reverse its directionality. MoS_2_/SiN delamination events are also present while applying pressure from the frontside of the membrane (*i.e.* causing membrane *indentation*), albeit different and reversible in character (Fig. 2). In comparison to MoS_2_ indentation experiments^37^ and bulging/indentation experiments with graphene^38–40^ this is not expected to cause delamination. In our case, we believe that the possible cause could be linked to substrate induced, local *wrinkling*. This effect has been seen in materials where the underlying substrate was compressed,^41–43^ where the 2D material locally detaches as it is energetically more favorable to loose adhesion than to conform to the deforming substrate curvature.^35^ We can further characterize the behaviour seen in Fig. 2 by doing streaming current and voltage measurements (Fig. 3 and 4). Streaming current measurements indicate the presence of liquid flow between the two sides of the membrane when the membrane is in a bulged state (Fig. 3a) most likely due to water permeating between the 2D material and the substrate, but in the indented state a streaming pathway is opened up a critical pressure is applied (≈2–3 bar, Fig. 3b). This critical pressure matches qualitatively the pressure values seen in ionic current measurements (Fig. 2b and c) and indicates an abrupt and temporary opening of a large pathway for fluid flow between the two sides of the membrane.

In order to clarify the nature of the ion transport, we opted to perform streaming potential measurements (Fig. 4c) using an electrometer grade buffer preamplifier. Streaming potential, unlike streaming current, does not in the first approximation depend on the effective size of the ionic channel (*i.e.* the geometry), and can be written as *V*_S_ = *ε*_r_*ε*_0_*ζP*/(*ση*) with *ε*_r_ the relative permittivity of water, *ε*_0_ the permittivity of vacuum, *ζ* the surface zeta potential, *P* the pressure gradient, *σ* the electrolyte solution conductivity and *η* the viscosity of the solution.^21,44,45^ In four samples showing the same qualitative behaviour as presented here we obtain comparable results *i.e.* reversible, local delamination or loss in adhesion that is caused directly by measurement conditions (high voltage or applied pressure). The reversible, impermanent character of this state is confirmed by comparing TEM images before and after measurements (see Fig. S4, ESI†). In the bulging case we note the presence of a streaming potential with zeta potential values ranging from 11 to 17 mV, typical for MoS_2_ pores and higher than the usual value of 8–9 mV we see in these conditions without any MoS_2_ present (pristine SiN_*x*_). This would indicate an occurrence of a water flowing through the MoS_2_ membrane and dragging solute ions in the surface double layer while in an indented/wrinkled state, possibly either through defects in the MoS_2_ or through a channel formed due to the normal force of the pressure lifting the MoS_2_ away from the aperture. The streaming potential in the indented case shows unstable and almost an order of magnitude larger values as pressure is applied. The pressure sweep protocol used in this work assumes the application of gradually increasing hydrostatic pressure with a pause at 0 bar in between the pressure steps (Fig. 4B and C). The observed, unstable variations in measured streaming potential do occur at each application of the pressure. This could suggest pressure-induced changes to MoS_2_ surface charges due to wrinkling – ion-conducting-wrinkle pathways changing and reshaping under each application of a hydrostatic pressure step.

## Conclusion

The use of pressure with suspended 2D materials on a thin membrane substrate is of interest as it allows probing the effects of lateral strain on the ion transport of nanopores in 2D materials^20,46^ as well as coupling of ion transport with fluid flow.^19,20^ Both these effects require a good understanding of the behaviour of such systems under mechanical and electrical strain. This work demonstrates how both permanent and temporary delamination of 2D materials can be induced by pressure and potential differences between the membrane. We demonstrate that the application of pressure pulses can change the adhesion and shape of delaminated 2D film wrinkles leading to unstable surface charges resulting in unexpected transmembrane streaming currents. We show a measurement methodology that allows to detect these adhesion issues and accurately identify them. The suspended 2D film wrinkling phenomenon needs to be taken into consideration while using pressure probes for wetting, bubble gating through applied hydrostatic pressure^21^ and probing ionic properties using pressurized setups.^20,30,47^ Although the large modulation of ionic transport due to partial delamination is similar to the case of pressure induced nanoparticle blockages^47^ and could be used to produce more robust mechanical pressure sensors, it is still far away from true mechanosensitivity where ion transport is modulated by mechanical stress directly modulating the energy barriers for single ion translocations.^46,48^ Our approach allows a better understanding of measurements with supported 2D materials, *i.e.* avoiding misinterpreting the measured data and could be used to probe how the electrical field and fluid flow at the nanoscale would influence membrane adhesion. Given the dynamic and volatile nature of the delamination events, the design and fabrication of nanofluidic devices with supported 2D films has to be revised to ensure stable and reliable device performance.

## Methods

Substrate preparation MoS_2_ was synthesized on a 3-inch sapphire substrate with a tube-furnace MOCVD setup using the liquid-promoter approach.^49,50^ Cleaned and annealed in air sapphire wafers were coated with sodium molybdate mixture and heated to 870 °C under ambient argon flow. After reaching the designated temperature the substrate was subjected to the flow of 12 sccm of molybdenum hexacarbonyl (MoCO_6_), 4 sccm of diethyl sulfide (DES), 4 sccm of hydrogen and 1 sccm of oxygen for 30 min. Synthesized MoS_2_ on sapphire was then cooled naturally under argon atmosphere. As-grown monolayer MoS_2_ was transferred onto holey Si/SiN membrane chips (Norcada) using wet transfer method^17,49^ and imaged in the transmission electron microscope (TEM) to confirm the successful fabrication of a suspended MoS_2_ membrane.

TEM imaging Before and after nanofluidic experiments suspended MoS_2_ membranes were imaged at ThermoFischer Talos F200S at 80 kV accelerating voltage. After the nanofluidic measurement, substrates were taken out of the flowcell and bathed several times in hot DI water to dissolve the remaining salts and minimize subsequent device contamination and salt crystallization. Substrates were then gently dried and imaged. All salt-cleaning steps were performed with caution to minimize the fluid flow and potential MoS_2_ delamination caused by that.

Nanofluidic measurements Samples used were in the form of 5 × 5 mm Si/SiN membrane devices mounted into a fluidic chamber as described previously and using the same measurement protocols.^20,21^ All liquids in touch with the sample were *in situ* degassed using a 925 μl Systec AF degassing chamber. Electrical measurements were done using a Zurich Instruments MFLI lock-in amplifier with the MF-DIG option using chlorinated Ag/Cl electrodes. Both DC and AC bias was applied using the signal output of the instrument, while the current through the sample was measured using the built in current to voltage converter. Streaming potential measurements were performed using an ultra-low input bias current (femtoampere level) electrometer grade buffer using the ADA4530 opamp. All electrical measurements done while sweeping the pressure were done after the pressure level has stabilized to at least 5% of the target value. In the case of DC current measurements, an additional wait time of 1 s was performed after the pressure settling.

We used 1 M KCl with 10 mM Tris buffered to pH 8 for all conductance measurements. All buffers were prepared using MiliQ grade water (18.2 MΩ cm^−1^). The conductivity of all solutions was checked before use with a Mettler–Toledo FiveEasy Plus. All solutions were filtered through a 20 nm filter before use (Whatman Anotop 25 plus).

## Author contributions

A. R., S. M., and M. M. conceived and designed the experiments; M. M. synthesized MoS_2_, M. T. performed thin film transfer; M. M. did nanofluidic measurements and TEM imaging, M. M. wrote the paper, with inputs from all authors; A. R and S. M. supervised the project; All authors discussed the results and commented on the manuscript.

## Conflicts of interest

There are no conflicts to declare.

## Supplementary Material

CP-024-D2CP02094G-s001
