# Supplementary material for: Stress induced delamination of suspended MoS2 in aqueous environments
Source: Phys Chem Chem Phys. 2022 Jul 29;24(33):19948–55. doi: 10.1039/d2cp02094g (PMC9500489; doi:10.1039/d2cp02094g)
Supplement: CP-024-D2CP02094G-s001 [file CP-024-D2CP02094G-s001.pdf]

Electronic Supplementary Material (ESI) for Physical Chemistry Chemical Physics.
This journal is © the Owner Societies 2022

Supplementary to Stress induced delamination of suspended MoS_2 in aqueous environments

Michal Macha[1*], Mukeshchand Thakur[1], Aleksandra Radenovic[1*] and Sanjin Marion[1,2*]

[1]Ecole Polytechnique Federale de Lausanne (EPFL), Lausanne, Switzerland.
[2]Current address: imec, Kapeldreef 75, B-3001 Leuven, Belgium.

*Corresponding author(s). E-mail(s): michal.macha@epfl.ch; aleksandra.radenovic@epfl.ch; sanjin.marion@imec.be;

List of Figures

S1 IV measurement of delaminating MoS_2 3
S2 Pressure-induced delamination 4
S3 HRTEM images of delaminating substrates before and after measurement 4
S4 HRTEM images of wrinkling substrate before and after measurement 5

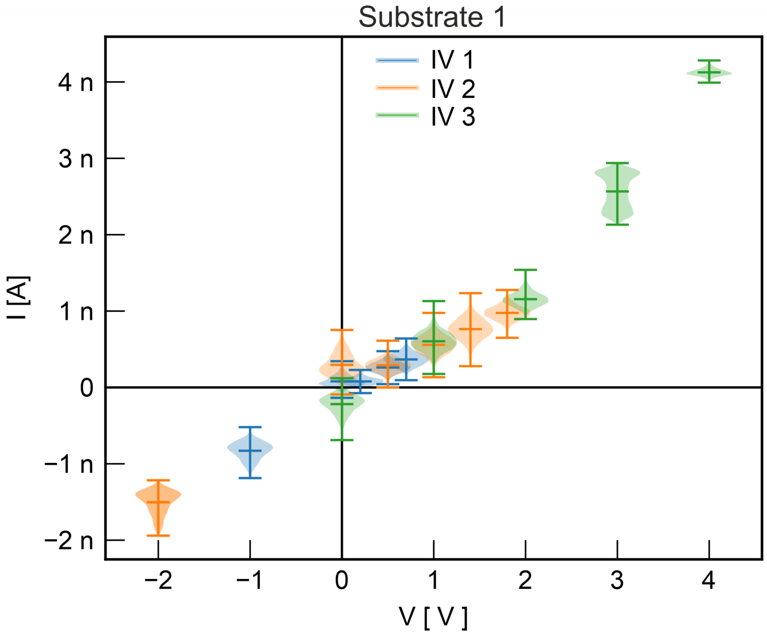

Fig. S1 IV measurement of delaminating MoS_2 Data extracted from ECR drilling procedure represented as an IV characteristic.

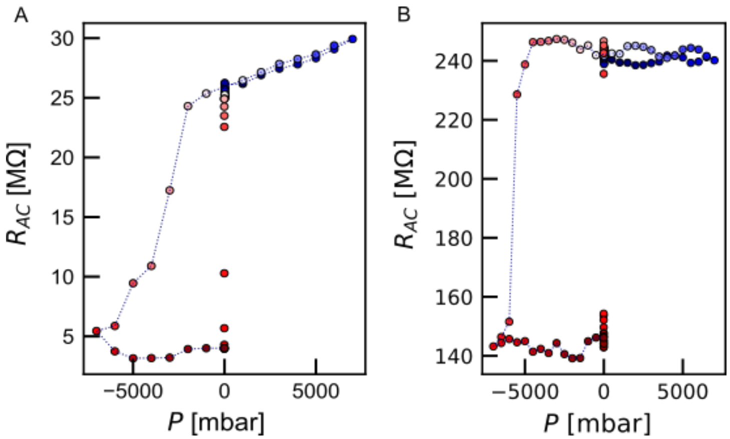

Fig. S2 Permanent, pressure-induced delamination. A pressure sweep measurement of delaminating MoS_2 film shown on two representative substrates (A) and (B). Membrane is swept with a pressure gradient (starting from 0 to 7 bar applied on the **frontside**, back to 0 and again to 7 bar applied on **backside** of the membrane and again back to 0 bar) under constant 100mV AC. Visible increase in ionic current occurs abruptly between 2 (A) and 5 (B) bar depending on the substrate. This indicates that the state of the membrane under no external applied force (voltage or pressure) has changed, supporting the scenario of irreversible delamination. Measurements were performed with the methodology described in the previous work using quasi-DC sinusoidal applied voltage[1, 2]

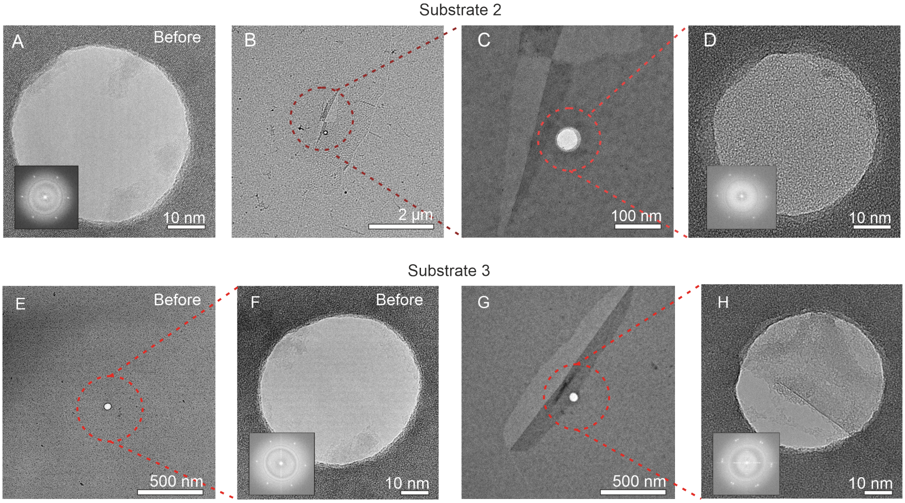

Fig. S3 HRTEM images of delaminating substrates before and after measurement. igh resolution TEM images of substrate 2 before (A) and after (B-D) showing visible delaminated material near nanopore viccinity. Similarily, substrate 3 before (E-F) and after (G-H) images show delamination occuring around the same area. All images are supported with FFT images (insets) which confirm the presence of MoS_2.

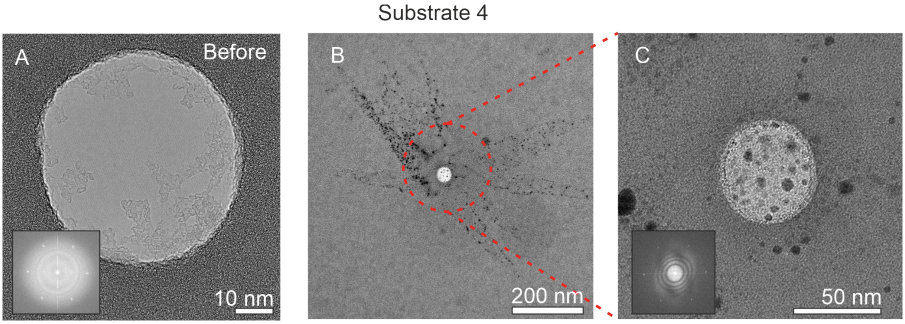

Fig. S4 HRTEM images of wrinkling substrate before and after measurementA high resolution TEM image of sustrate 4 before (A) and after (B-C) nanofluidic measurements with MoS_2 remaining suspended over the membrane aperture. Insets represent FFT images with MoS_2 pattern, confirming its presence on the suspended area.

References

[1] Marion, S., Macha, M., Davis, S.J., Chernev, A., Radenovic, A.: Wetting of nanopores probed with pressure. Physical Chemistry Chemical Physics **23**(8), 4975–4987 (2021). https://doi.org/10.1039/D1CP00253H

[2] Davis, S.J., Macha, M., Chernev, A., Huang, D.M., Radenovic, A., Marion, S.: Pressure-Induced Enlargement and Ionic Current Rectification in Symmetric Nanopores. Nano Letters (2020).
